# YOLO-VOLO-LS: A Novel Method for Variety Identification of Early Lettuce Seedlings

**DOI:** 10.3389/fpls.2022.806878

**Published:** 2022-02-24

**Authors:** Pan Zhang, Daoliang Li

**Affiliations:** ^1^National Innovation Center for Digital Fishery, China Agricultural University, Beijing, China; ^2^Beijing Engineering and Technology Research Centre for Internet of Things in Agriculture, China Agriculture University, Beijing, China; ^3^China-EU Center for Information and Communication Technologies in Agriculture, China Agriculture University, Beijing, China; ^4^Key Laboratory of Agricultural Information Acquisition Technology, Ministry of Agriculture, China Agriculture University, Beijing, China; ^5^College of Information and Electrical Engineering, China Agricultural University, Beijing, China

**Keywords:** hydroponic crops, greenhouse, deep learning, detection, classification, multiple growth stages

## Abstract

Accurate identification of crop varieties is an important aspect of smart agriculture, which is not only essential for the management of later crop differences, but also has a significant effect on unmanned operations in planting scenarios such as facility greenhouses. In this study, five kinds of lettuce under the cultivation conditions of greenhouses were used as the research object, and a classification model of lettuce varieties with multiple growth stages was established. First of all, we used the-state-of-the-art method VOLO-D1 to establish a variety classification model for the 7 growth stages of the entire growth process. The results found that the performance of the lettuce variety classification model in the SP stage needs to be improved, but the classification effect of the model at other stages is close to 100%; Secondly, based on the challenges of the SP stage dataset, we combined the advantages of the target detection mechanism and the target classification mechanism, innovatively proposed a new method of variety identification for the SP stage, called YOLO-VOLO-LS. Finally, we used this method to model and analyze the classification of lettuce varieties in the SP stage. The result shows that the method can achieve excellent results of 95.961, 93.452, 96.059, 96.014, 96.039 in Val-acc, Test-acc, Recall, Precision, F1-score, respectively. Therefore, the method proposed in this study has a certain reference value for the accurate identification of varieties in the early growth stage of crops.

## Introduction

With the integration of modern information technology such as artificial intelligence, big data, and the Internet of Things with agricultural development, smart agriculture has become the inevitable direction of agricultural development (Kussul et al., 2017; Mei et al., 2018). As one of the important contents of the development of smart agriculture, the intelligent identification and classification of crop varieties is crucial to the management of the differences in later crop production (Suh et al., 2018; Khamparia et al., 2020). In addition, there are certain differences between different varieties of the same crop in terms of growth cycle, fertilizer requirements, light requirements, heat resistance, cold resistance, etc. (Yalcin and Razavi, 2016). If they are not distinguished and identified, they will face many problems in later production management. The traditional crop variety identification process mostly relies on human identification by experts and planters, which is time-consuming, laborious and inefficient (Sun et al., 2017), and it is difficult to automatically connect tasks such as intelligent irrigation, fertilization, grading, sorting, packaging, and harvesting in the planting process. Therefore, there is an urgent need for an intelligent identification method to automate the identification of crop varieties in order to realize the unmanned connection of related tasks in the whole production process.

The advent of artificial intelligence provides a strong technical support for the intelligent identification of crop varieties (Tan et al., 2020). Image processing, machine learning, and deep learning have been continuously integrated and applied with agricultural research (Kim et al., 2018; Kaya et al., 2019). As far as crop varieties recognition is concerned, traditional image processing methods have been applied earlier (Dharwadkar et al., 2017; Tiwari, 2020). However, traditional image processing methods require researchers to manually design and extract features, such as the color, shape, and texture of crop leaves. There is a certain degree of blindness in this process (Bhosle and Musande, 2019). At the same time, based on the manually extracted feature data, neural networks composed of neurons, such as multi-layer perceptron, are used for model construction (Zhang et al., 2020). Many parameters need to be manually adjusted in the modeling process, and the model is easy to over fit, which increases the difficulty of model construction (Yoosefzadeh-Najafabadi et al., 2021). With the rise of artificial intelligence algorithms such as deep learning, it has gradually made breakthroughs in progress in the fields of computer vision, image classification, target detection, target segmentation, and speech recognition (Yalcin and Razavi, 2016). Deep learning is a new field of machine learning, which automatically analyzes data and extracts features by simulating the brain (Tóth et al., 2016; Khamparia et al., 2020). In data processing, a neural network for the target task is established through the basic CNN network, and the characteristics of the input data (such as color, texture, shape, etc.) are extracted layer by layer, and a good mapping relationship from the underlying signal to the high-level semantics is established (Dileep and Pournami, 2019). Therefore, deep learning may have more advantages in crop varieties identification.

In recent years, research on the recognition of crop varieties or types based on deep learning methods are mostly in field planting scenarios (Grinblat et al., 2016; Teimouri et al., 2019). On the one hand, for agronomists and agricultural institutions specializing in land management, it is very important to fully understand the specific conditions of land use and dynamically monitor crop planting within a certain period of time. For example, Mazzia et al. (2020) used remote sensing technology to obtain multi-temporal sentinel-2 images in central and northern Italy and combined recurrent neural network (RNN) and convolutional neural network (CNN) to propose a pixel-based LC&CC deep learning model for the region’s type identification of agricultural crops. By comparing traditional support vector machine, random forest, and other methods, the accuracy of the proposed LC&CC deep learning method can reach 96.5%. In order to achieve accurate, automatic, and rapid crop mapping, Sun et al. (2020) built a deep neural network classification model based on historical crop maps and ground measurement data in North Dakota, and a high-quality map of seasonal crops was generated from Landsat images of North Dakota. At the same time, when the model was applied to new images, accurate results were obtained on major crops such as corn, soybeans, barley, spring wheat, dried beans, sugar beets, and alfalfa. For the problem of spectral similarity between different plants in the same family and genus, Zhang et al. (2020) used an improved three-dimensional CNN to build a tree species classification model based on a remote sensing data set with rich spectral and spatial characteristics. The results show that this method can reach 93.14% accuracy. In order to better capture the temporal and spatial characteristics of crop classification, Gadiraju et al. (2020) proposed a multi-modal deep learning method that combines spatial spectrum and phenological characteristics. Among them, the spatial characteristics of the image are obtained through CNN, and the phenological characteristics of the image are obtained through LSTM. The results show that this method can reduce the error by 60%. In addition, the accurate identification of agricultural products varieties is not only an urgent need of dealers, but also an urgent need of product processing enterprises and consumers. Rong et al. (2020) collected the visible and near-infrared spectrum data of five peach varieties between 350 and 820 nm, and then constructed a one-dimensional CNN to identify peach varieties with an accuracy of 94.4%. Liu et al. (2020) used machine learning and computer vision technology to classify 7 kinds of chrysanthemum tea. Compared with traditional morphological feature extraction (90%), the classification performance of deep neural network is better (96%). Liu et al. (2019) used VGG16 and ResNet50 to identify chrysanthemum varieties, which further proved that the deep learning method applied to variety recognition research has the advantages of strong recognition performance and fast recognition speed, which is a breakthrough in horticultural science. Van Hieu and Hien (2020) obtained images of 109 Vietnamese plants through the Vietnam Encyclopedia, and then used MobileNetV2, VGG16 and other methods to construct classification models. The results showed that MobilenetV2 has the highest recognition rate of 83.9%. Bisen (2021) built a recognition and classification system for different crops based on leaves, and extracted leaf features through a CNN, and finally got an accuracy of 93.75%. Nkemelu et al. (2018) compared the classification performance of two traditional methods and CNNs based on image data sets of 960 plant species at 12 different growth stages. The results show that reasonable use of CNNs can achieve ideal classification results. Grinblat et al. (2016) used a deep CNN to build a crop classification model based on the leaf vein patterns of three bean crops, and the results showed that the effect of the leaf vein-based crop classification model has been significantly improved. At the same time, it has been proved that increasing the network depth can further improve the effect of the model. Tan et al. (2020) also constructed a crop model based on the vein characteristics of plants, and achieved good results. At the same time, the effectiveness of leaf vein characteristics in the process of plant classification was also proved by Lee et al. (2017). In large-scale plant species identification and classification, in order to improve the accuracy and computational efficiency of plant species identification, Zhang H. et al. (2018) proposed a path-based tree classifier deep learning method. The classification is carried out in a detailed hierarchical structure, and the effect is significantly improved. Similarly, some researchers have used MaskRCNN, AlexNet, CNN and other methods to identify each varieties of bananas (Le et al., 2019), grapes (Pereira et al., 2019), lemons (Alzamily et al., 2019) and medicinal materials (Dileep and Pournami, 2019; Duong-Trung et al., 2019), and achieved good results. On the other hand, in some countries where small farms are the main planting model, there are more small-scale land, dense intercropping, and diverse crop types. Chew et al. (2020) obtained image data through drones and built a recognition model for bananas, corn, beans and other crops based on VGG, and achieved good results. However, in the case of crop intercropping, there are certain limitations in the recognition accuracy of different crops. Synthetic aperture radar data also has certain advantages in remote sensing crop recognition. Teimouri et al. (2019) proposed a new method—FCN-LSTM by combining full convolutional network (FCN) and long short-term memory network (LSTM). This method has been applied to radar data to construct a remote sensing crop classification model, and the results show that the accuracy of the method in the classification of 8 crops based on pixel recognition exceeds 86%.

Based on the results of the above research, although there have been studies on species or type identification for some crops, most of the application scenarios are field planting, and a small number of application scenarios are gardening, and there are few scenarios such as greenhouses (Zhang H. et al., 2018; Teimouri et al., 2019; Gadiraju et al., 2020). The devices used mainly include spectroscopy and digital cameras (Zhang et al., 2020; Bisen, 2021). Among them, spectroscopy equipment is expensive, and it is mostly used in large-scale planting scenarios (Zhang et al., 2020). Digital cameras are relatively cheap, and can meet the needs of low-altitude remote sensing and greenhouse scenes (Bisen, 2021). However, due to the limitations of the greenhouse space and structure, there are certain risks in carrying the camera on the drone equipment, but carrying the camera on the mobile robot can achieve most of the greenhouse agricultural production tasks (Zhang et al., 2019). In addition, most of the above studies directly use deep learning classification methods (such as AlexNet, VGG, ResNet, etc.) to identify the varieties or types of different crops (Ghazi et al., 2016). In the greenhouse scenario, the initial growth of most crops is relatively small and the background features account for a large proportion. In this case, the direct classification of varieties or types of crops may cause a certain loss of accuracy. However, by combining target detection and classification, there may be unexpected results.

Therefore, this study conducted the identification of lettuce varieties at different growth stages for 5 kinds of greenhouse hydroponic lettuce under 6 nitrogen treatments. The classification method and target detection method are used for crop variety recognition, and the two are combined for lettuce variety recognition to explore the improvement of model performance. The novel contributions of this article are concluded as follows: (1) We constructed 7 lettuce classification datasets under different growth stages and different nitrogen treatments, and used the dataset to study the classification of lettuce varieties under the influence of multiple factors. (2) We used classification-based and detection-based methods for lettuce variety recognition, and compared the performance of lettuce variety recognition models at different growth stages. (3) We propose a lettuce variety recognition method called YOLO-VOLO-LS, which combines classification mechanism and detection mechanism, and discuss its challenges and opportunities in future application.

## Materials and Methods

### Experimental Field

**FIGURE 1:**
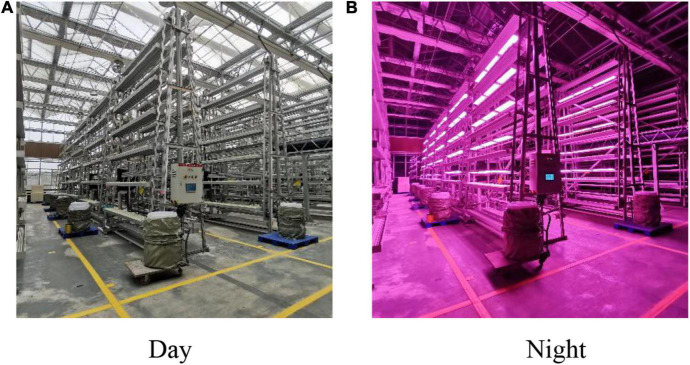
Greenhouse cultivation environment. **(A)** Represents the cultivation environment under daytime conditions, and **(B)** represents the cultivation environment under night conditions.

**FIGURE 2:**
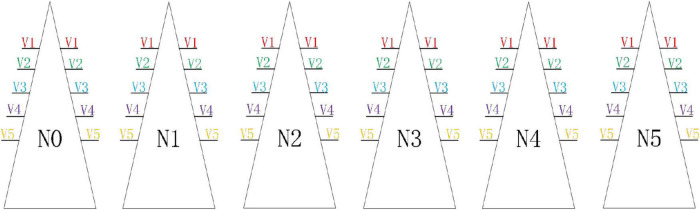
Distribution of lettuce in stereoscopic cultivation racks of 5 varieties. There were a total of 6 cultivation racks, and each cultivation rack was equipped with a nitrogen concentration treatment, and the same variety of lettuce was symmetrically transplanted at the same position on both sides of each cultivation rack.

This experiment was conducted in the glass greenhouse of the Factory Agricultural Research and Development Center of Chongqing Academy of Agricultural Sciences from March to May of 2021 (Figure 1). Five varieties of lettuce were selected for the experiment, namely, Selected Italian (V1), Small cream green (V2), Rosa green (V3), Badawiya (V4), and Boston cream (V5). In the seedling stage, we selected the full-grained lettuce seeds and placed them in the seedling cotton with 100 grooves for seeding, with one seed in each groove. The temperature was controlled at 23–26°C, the air humidity was 60–70%, the seedling cotton was kept moist, and the halogen lamp was used to supplement the light after germination. After 20 days of seedlings, we transplanted 5 varieties of lettuce to 6 cyclically rotating stereoscopic cultivation racks. Each cultivation rack was set to 0, 33, 66, 99, 132, and 165% according to the nitrogen concentration in the standard nutrient solution. Five slots were designated on both sides of each cultivating rack to cultivate a specific variety of lettuce. Each slot can grow 81 lettuces, and the two sides of the stereoscopic cultivating rack are correspondingly placed with the same variety of lettuce (Figure 2). The growth process of lettuce adopted the way of hanging roots, and the nutrient solution was changed every 3 days. Normal greenhouse cultivation and management of lettuce were conducted, and no pesticides and hormones were applied.

### Image Data Acquisition

**FIGURE 3:**
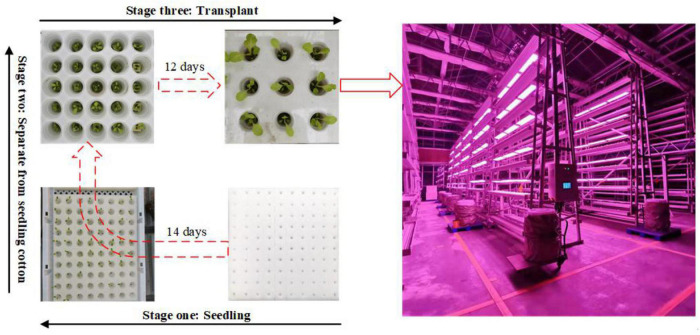
The whole process from seedling to transplanting of the lettuce. The whole process mainly included 3 stages, namely seedling, separating from seedling cotton, and transplanting.

The whole process from seedling to transplanting of lettuce in this experiment mainly included three stages: seedling (SL), separate planting (SP), and transplanting (TP), as shown in Figure 3.

First, after we separated the five varieties of lettuce from the seeding cotton, we collected data every other day for the next 12 days. We conducted collections six times in total, and 160 images of each lettuce are collected each time. Second, after the five varieties of lettuce seedlings were transplanted to the stereo cultivation rack, we carried out image data acquisition every 5 days. Each data collection mainly acquired 50 lettuce images of different varieties and different nitrogen nutrient gradient treatments, each of which had six nitrogen treatments, for a total of 300 images. The data collection time was 9:00–17:00, and in order to ensure the consistency of data collection, the camera was kept perpendicular to the plane of the planting slots and at a distance of 40 cm during the collection process.

### Data Pre-processing

Based on the aforementioned data acquisition process, the number of data acquired on Days 1, 6, 12, 18, 24, and 30 was only 300, which cannot meet the data volume requirements for deep learning training. Based on the principle of cross-validation, we first randomly divided the lettuce data set of each variety according to the ratio of 6:2:2. Second, we performed data enhancement through rotation, flipping, and contrast adjustment, 23 times. Finally, the dataset volume of the training, validation and test for each growth stage of each variety after data enhancement was 4,140, 1,380, and 1,380, respectively.

**FIGURE 4:**
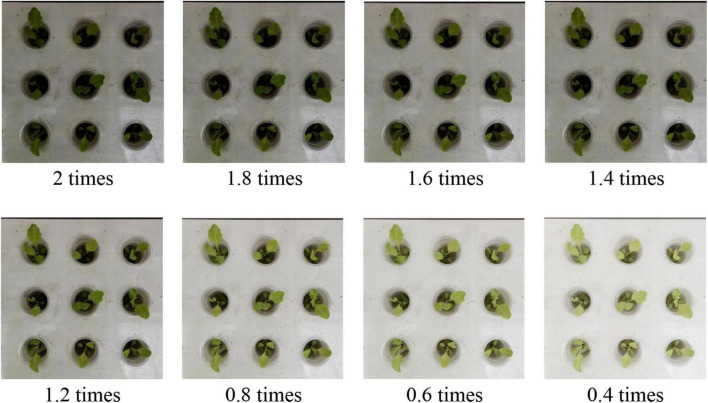
Data enhancement based on contrast adjustment. The adjustment of the image contrast is realized by the gamma adjustment method. When gamma > 1, the new image is darker than the original image. If gamma < 1, the new image is brighter than the original image.

In addition, in order to ensure that our model can achieve accurate classification of lettuce varieties in various greenhouse scenarios, we used a contrast adjustment method in data enhancement to improve the richness of data. Specifically, this is as shown in Figure 4.

### YOLO-VOLO

**FIGURE 5:**
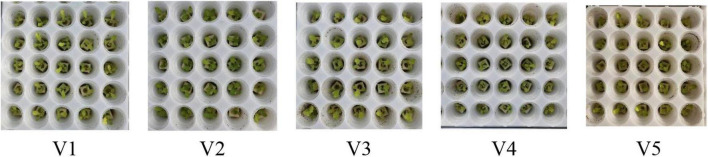
Five kinds of lettuce involved in this study. Among them, Italian (V1), Small cream green (V2), Rosa green (V3), Badawiya (V4), and Boston cream (V5).

**FIGURE 6:**
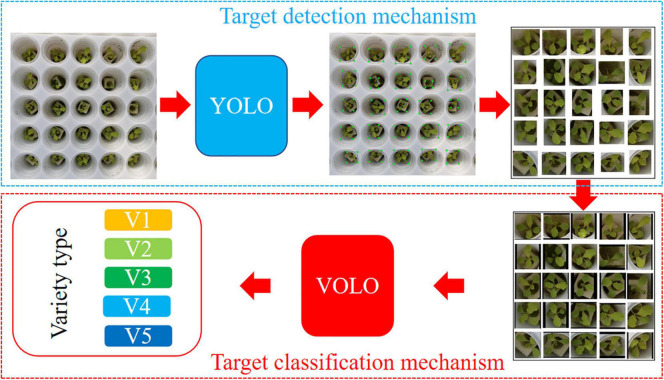
Data processing process of the YOLO-VOLO model. The input RGB image is first detected and cropped by the YOLO algorithm, and then input into the VOLO algorithm for lettuce species identification after boundary padding, and finally the category of the lettuce species is output.

In this study, the recognition of the five lettuce varieties in **SP** had problems of strong background interference, high similarity, and difficulty in classification (Figure 5). We propose a new method called YOLO-VOLO to identify lettuce varieties in the SP stage by combining target detection and target classification mechanisms (Figure 6), so as to achieve a relatively ideal classification effect.

#### YOLO-v5 for Target Cutting

First, we use the LabelImg software to set the label for the five lettuce datasets of **SP** as Plant. Then, YOLO-v5 (Jia et al., 2021; Kasper-eulaers et al., 2021; Liu W. et al., 2021), the most advanced algorithm of Yolo series, was used to detect the lettuce plant in **SP**, namely separating from seedling cotton. After training an object detection model independently in **SP**, we cut the lettuce plants according to the coordinates of the location of each plant predicted by the target detection model. Due to the differences in the growth of each lettuce plant, the cropped objects are of different sizes. In order to ensure that each object maintains the original image aspect ratio during the later model training, we used the boundary padding method to perform data preprocessing (Figure 6A), according to the characteristics of the image datasets of **SP** of lettuce, and considering the requirements of image resolution, GPU memory and accurate detection. We put the image datasets of the Stage two into the neural network for training (the image resolution is 384 * 384), where three different sizes of detection head, 52*52, 26*26, and 13*13 are used to output the results including the lettuce’s position information, category information and confidence.

#### VOLO for Target Classification

Based on the lettuce images obtained from the processing in Section “YOLO-v5 for target cutting,” we used the current state-of-the-art target recognition algorithm VOLO to classify the five lettuce images. VOLO is a network structure with two independent stage (Yuan et al., 2021). First, a stack of Outlookers that generates a fine-grained token representation constitutes the first independent stage. Secondly, a second independent stage is formed to aggregate global information by deploying a series of transformer blocks. At the beginning of each stage, a patch embedding module is used to map the input to the marked representation of the design shape.

Outlooker is a newest simple and lightweight attention mechanism module, which can effectively use fine-level information to enrich token representation. In addition, Outlooker has made certain innovations in generating attention for token aggregation, allowing the model to efficiently encode fine-level information. In particular, an effective linear mapping method can directly infer the mechanism of gathering surrounding tokens from the characteristics of anchored tokens, thereby avoiding expensive point product attention calculations. The Outlooker is composed of the outlook attention layer used for spatial information encoding and the multi-layer perceptron (MLP) used for information interaction between channels. Given a sequence of input *C*−dim token representations *X* ∈ *R*^*H*×*W*×*C*^, Outlooker can be represent as follows:


(1) $$\tilde{X}=O\,u\,t\,l\,o\,o\,k\,A\,t\,t\,\left(L\,N\,\left(X\right)\right)+X$$

(2) $$Z=M\,L\,P\,\left(L\,N\,\left(\tilde{X}\right)\right)+\tilde{X}$$

Where LN refer to LayerNorm (Liu F. et al., 2021).

Among them, Outlook attention is efficient, easy, and simple to implement. The main characteristics are: (1) The features at each spatial location are sufficiently representative to generate attention weights for local aggregation of neighboring features; (2) Dense local spatial aggregation can effectively encode fine-level information.

**FIGURE 7:**
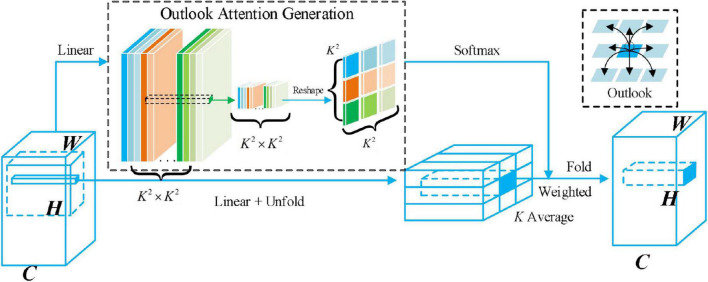
Illustration of outlook attention. The outlook attention matrix for a local window of size K*K can be simply generated from the center token with a linear layer followed by a reshape operation. The attention weights are generated from the center token within the window and act on the neighbor tokens and itself.

As we can see from Figure 7, for a partial window with a size of K × K, a linear layer can be simply generated from the central token, and a reshaping operation (highlighted by the green box) can then be performed. Since the attention weight is generated by the center mark in the window and acts on the adjacent mark and itself (highlighted by the black box), we call these operations Outlook attention. For each spatial location (*i*,*j*), Outlook attention calculates the similarity between it and all neighboring features in a local window of size K*K centered on (*i*,*j*). Unlike self-attention, which requires query key matrix multiplication to calculate attention (i.e., $S\,o\,f\,t\,m\,a\,x\,\left(Q^{T}\,K/\sqrt{d}\right)$), Outlook attention simplifies this process through a reshaping operation.

Normally, when we give input*X*, we first project each *C*−*dim* token, and then use two linear weights *W*_*A*_ ∈ *R*^*C*×*K*^4^^ and *W*_*V*_ ∈ *R*^*C*×*C*^, respectively, and the projection is the outlook weight *A* ∈ *R*^*H*×*W*×*K*^4^^ and the value represents *V* ∈ *R*^*H*×*W*×*C*^. *V*_Δ*i*,*j*_ ∈ *R*^*C*×*K*^2^^ denotes all the values in the local window centered on (*i*,*j*), i.e.,


(3) $$V_{\Delta_{i,j}}=\left\{V_{i+p-\left|\frac{K}{2}\right|,j+q-\left|\frac{K}{2}\right|}\right\},0\leq p,q<K$$

**Outlook attention:** The outlook weight of location (*i*,*j*) is directly used as the attention weight of value aggregation, which is reshaped into $\overset{\wedge }{\underset{i,j}{A}}\in M\,a\,t\,M\,u\,l\,\left(S\,o\,f\,t\,m\,a\,x\,\left(\overset{\wedge }{\underset{i,j}{A}}\right),V_{\Delta_{i,j}}\right)$, and then the *Softmax* function is used. Therefore, the value projection process can be written as:


(4) $$Y_{\Delta_{i,j}}=\sum_{0\leq m,n<K}Y_{\Delta_{i+m-\left|\frac{K}{2}\right|,j+n-\left|\frac{K}{2}\right|}}^{i,j}$$

**Dense aggregation:** Outlook attention intensively gathers the expected value representatives, summing up weighted values from the same position of different local windows to get the output:


(5) $$\overset{∼}{\underset{i,j}{Y}}=\sum_{0\leq m,n<K}Y_{\Delta_{i+m-\left|\frac{K}{2}\right|,j+n-\left|\frac{K}{2}\right|}}^{i,j}$$

The implementation of the multi-head outlook attention mechanism is as follows: Assuming that the number of heads is set to *N*, we only need to adjust the weight shape of *W*_*A*_ to make*W*_*A*_ ∈ *R*^*C*×*N*∙*K*^4^^. Then the foreground weight and value embedding are evenly divided into *N* segments to obtain *A*_*n*_ ∈ *R*^*H*×*W*×*K*^4^^ and *V*_*n*_ ∈ *R*^*H*×*W*×*C*_N_^, {*n* = 1, 2,…, *N*}, where the size of each head of *C*N** satisfies *C*N** × *N* = *C*. For each (*A*n**, *V*n**) pair, the foreground attention is calculated separately, and then connected as the output of the multi-head foreground attention. In our study, due to the limitation of computer hardware (GPU memory only supports VOLO-D1), we mainly used VOLO-D1 to conduct the lettuce variety identification of **SP** with 384-size input images. For detailed information about several variants of the VOLO algorithm (see Table 1).

**TABLE 1:** Architecture information of different variants of VOLO.

Specification	VOLO-D1	VOLO-D2	VOLO-D3	VOLO-D4	VOLO-D5
Patch embedding	**8 × 8**	8 × 8	8 × 8	8 × 8	8 × 8
Stage 1 (28 × 28)	**[Head:6, stide:2 Kernel: 3 × 3 Mlp:3, dim:192] × 4**	[Head:8, stide:2 Kernel: 3 × 3 Mlp:3, dim:256] × 6	[Head:8, stide:2 Kernel: 3 × 3 Mlp:3, dim:256] × 8	[Head:12, stide:2 Kernel: 3 × 3 Mlp:3, dim:384] × 8	[Head:12, stide:2 Kernel: 3 × 3 Mlp:4, dim:384] × 12
Patch embedding	**2 × 2**	2 × 2	2 × 2	2 × 2	2 × 2
Stage 2 (14 × 14)	**[#heads:12, Mlp:3, dim:384] × 14**	[#heads:16, Mlp:3, dim:512] × 18	[#heads:16, Mlp:3, dim:512] × 28	[#heads:16, Mlp:3, dim:768] × 28	[#heads:16, Mlp:4, dim:768] × 36
Total layers	**18**	24	36	36	48
Parameters	**26.6M**	58.7M	86.3M	193M	296M

*Bold highlights the specific method used in the manuscript.*

After constructing the lettuce variety identification model at the SP stage by using classification and detection methods, respectively, we thought about how to avoid background interference and the similarity between plants at the same time. Finally, we propose a method, namely YOLO-VOLO. The core idea of this method is to combine the advantages of detection and classification methods to simplify the problem of plant population classification into an individual classification problem.

**Step 1**: we take advantage of the strong detection ability of YOLO-V5 to cut out different varieties of lettuce plants. Because there are individual growth differences between different plants, we use border filling to ensure that each picture maintains the original horizontal and vertical ratio.

**Step 2**: we take advantage of the strong classification ability of VOLO-D1 to construct a classification model for the individual plant images obtained in the **Step 1**.

### Result Evaluation

The verification of model performance is very important. When the data amount of various samples in the training dataset is evenly distributed, the commonly used *Accuracy* is used to evaluate the performance of the model; when the data amount of various samples in the training dataset is not uniformly distributed, it is necessary to refer to other indicators to evaluate the model performance, such as *Precision*, *Recall*, and *F1-Score*. The specific definitions are as follows:

***Accuracy***: This is defined as the ratio of correctly classified images to the total number of lettuce images.


(6) $$A\,c\,c\,u\,r\,a\,c\,y=\frac{T\,P+T\,N}{T\,P+F\,N+F\,P+T\,N}\times 100\%$$

***Precision***: This is defined as the average of the total number of images of correctly identified lettuce varieties and the total number of images of correctly and incorrectly identified lettuce varieties.


(7) $$P\,r\,e\,c\,i\,s\,i\,o\,n=\frac{T\,P}{T\,P+F\,P}\times 100\%$$

***Recall***: This is defined as the average of the images of correctly identified varieties of lettuce and the total number of correct and undetected images.


(8) $$R\,e\,c\,a\,l\,l=\frac{T\,P}{T\,P+F\,N}$$

***F1-score***: This is defined as the weighted average of Precision and Recall.


(9) $$F\,1-S\,c\,o\,r\,e=\frac{2\times R\,e\,c\,a\,l\,l\times P\,r\,e\,c\,i\,s\,i\,o\,n}{R\,e\,c\,a\,l\,l+P\,r\,e\,c\,i\,s\,i\,o\,n}$$

where *TP, FP, FN*, and *TN* represent true positive, false positive, false negative and true negative, respectively.

## Results

### Variety Recognition of Lettuce in Multiple Growth Stages Based on VOLO-D1

In order to explore the changing laws of lettuce variety identification at different growth stages, the state-of-the-art target recognition method VOLO was used to conduct a study on the variety identification of five lettuce at different growth stages. After trying different VOLO pre-training models, VOLO-D1 was finally selected as the main research method due to the limitation of computer hardware (insufficient GPU memory). The Accuracy, Recall, Precision, and F1-score are used as validation indicators to compare the classification models between different growth stages. The specific results are shown in Table 2.

**TABLE 2:** Classification model results of different growth stages of lettuce based on VOLO-D1.

Class	Image-size	Train-acc	Val-acc	Test-acc	Recall	Precision	F1-score
**SP**	**384**	**99.661**	**81.970**	**78.381**	**82.946**	**85.902**	**84.398**
Day 1	384	99.999	100	100	100	100	100
Day 6	384	99.999	100	100	100	100	100
Day 12	384	99.920	100	100	100	100	100
Day 18	384	99.981	100	100	99.783	100	99.889
Day 24	384	99.999	100	100	100	100	100
Day 30	384	99.999	100	100	100	100	100

*Bold highlights the results of model comparison.*

The results show that the performance of the VOLO-D1 method in the growth stage model, except the SP stage, is close to 100%, while the model accuracy in the SP stage is only 78.381. After analysis, it is found that the dataset at the SP stage has problems such as small plant targets and large background interference, which is not conducive to accurate identification of lettuce varieties. Therefore, the classification performance of the SP stage model needs to be further improved and optimized.

### Variety Recognition of Lettuce During SP Period Based on YOLO-v5

**FIGURE 8:**
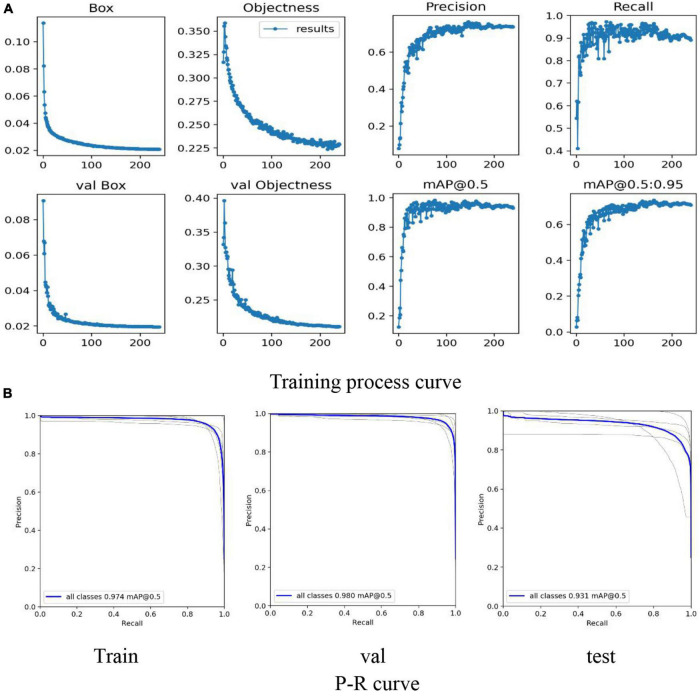
Classification model training process based on YOLO-V5. **(A)** Mainly reflects the Loss, Precision, Recall, mAP curves in the training process and the Loss curve in the verification process. **(B)** Mainly reflects the P-R curve of training, testing and verification to judge the pros and cons of the model.

Aiming at the challenges of the SP stage dataset, the feasibility of identifying lettuce varieties through the target detection method was explored. The most advanced algorithm YOLO-v5 of the current YOLO series was used to detect and classify 5 lettuce varieties in the SP stage (as shown in Figure 8), the input image size remains consistent with the VOLO-D1(384*384). The specific results are shown in Table 3.

**TABLE 3:** Classification model results of SP stage of lettuce based on YOLO-v5.

Class	Image-size	Recall	Precision	F1-score	mAP@0.5	mAP@0.5:0.95
Train	384	0.976	0.799	0.879	0.974	0.719
Val	384	0.971	0.760	0.853	0.980	0.740
Test	384	**0.892**	**0.737**	**0.807**	0.931	0.709

*Bold highlights the results of model comparison.*

By comparing the result of YOLO-v5 and VOLO-D1, the results show that the F1-score of the two are relatively close (0.879 and 0.844). At the same time, YOLO-v5 is better than VOLO-D1 on the F1-score, which to a certain extent shows that the classification performance of lettuce varieties can be improved by removing background interference or increasing the number of training targets.

As we can see from Figure 8, the results show that in the training and verification process of the model, all the curves have converged, eliminating the possibility of model overfitting.

### Variety Recognition of Lettuce During SP Period With a Fusion of YOLO and VOLO

Based on the foregoing attempts, the results prove that a single target classification and target detection method is not the best choice. Therefore, we explore the feasibility of the YOLO-VOLO-LS method proposed in this study by trying to combine the advantages of target detection and target classification algorithms.

**FIGURE 9:**
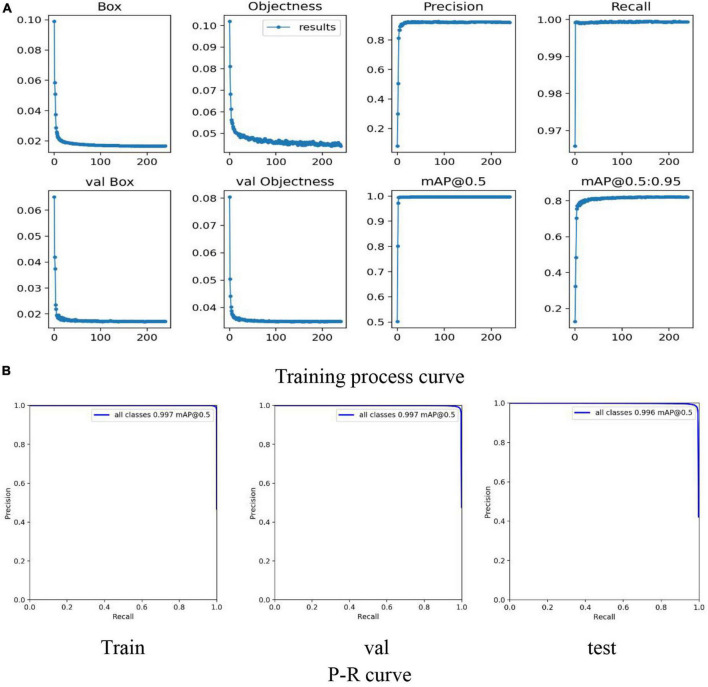
Detection model training process based on YOLO-V5. **(A)** Mainly reflects the Loss, Precision, Recall, mAP curves in the training process and the Loss curve in the verification process. **(B)** Mainly reflects the P-R curve of training, testing and verification to judge the pros and cons of the model.

The first step is to use the powerful detection capabilities of YOLO-V5 to uniformly modify the individual labels of the five varieties of lettuce plants to “plant,” and then train the lettuce detection model. According to the lettuce coordinates output by the trained model, the lettuce plants of different varieties are cut, respectively. Due to the individual growth differences between different plants, we use border padding to ensure that each image maintains the original aspect ratio. The specific results are shown in Table 4 and Figure 9.

**TABLE 4:** Detection model results of SP stage of lettuce based on YOLO-v5.

Class	Image-size	Recall	Precision	F1-score	mAP@0.5	mAP@0.5:0.95
Train	384	0.999	0.919	0.957	0.997	0.821
Val	384	0.997	0.936	0.966	0.997	0.743
Test	384	**0.995**	**0.934**	**0.964**	**0.996**	**0.694**

*Bold highlights the results of model comparison.*

By comparing Table 4 with Table 3, the result shows that when the 5-class detection task is simplified to a single-class detection task, the Recall, Precision, F1-score, mAP@0.5 are significantly improved. Among them, Recall, Precision, F1-score, and Map@0.5 have improved 0.103, 0.197, 0.157, and 0.065, respectively. Compared with the 5-class detection problem, the single-class detection task does not have the influence of problems such as the similarity between classes, so the model performance is excellent.

As shown in Figure 9A, all the curves in the training process have good convergence (faster convergence), and the curve is smooth, with almost no oscillations. As shown in Figure 9B, the result shows that mAP@0.5 has a significant performance improvement, and all reach more than 0.99. Therefore, the lettuce target detection model based on YOLO-V5 can accurately detect the location of the plant, provide accurate coordinate information for the cutting process, and obtain the lettuce individual plant dataset required by the subsequent classification model.

In the second step, using the individual lettuce dataset obtained from the first steps, we further explore the classification performance of the VOLO-D1 method on this dataset. Because in the SP stage there are certain similarities between different varieties of lettuce plants, and in order to further improve the performance of the model, we used the label smoothing (LS) trick to further optimize the YOLO-VOLO model. In addition, in order to prove that reducing background interference has a greater contribution to the model than increasing the amount of data, we performed a 5*5 slicing operation on the original dataset to ensure that the amount of data in the method proposed in this study is consistent, and then use the VOLO-LS method to perform Model training. The specific results are shown in Table 5.

**TABLE 5:** Classification model results of SP stage of lettuce based on YOLO-VOLO.

Class	Image-size	Train-acc	Val-acc	Test-acc	Recall	Precision	F1-score
YOLO-VOLO	384	99.184	**92.547**	**92.001**	**92.785**	**93.032**	**92.908**
Slice-VOLO-LS	384	99.365	87.695	87.682	87.925	88.324	88.124
YOLO-VOLO-LS	384	99.654	**95.961**	**93.452**	**96.059**	**96.014**	**96.039**

*Bold highlights the results of model comparison.*

By comparing Tables 4–6, the F1-score was selected as the indicator to comprehensively evaluate the performance of the model. The results show that YOLO-VOLO-LS is better than VOLO-D1 and YOLO-V5 by 11.641, 15.339 on F1-score, respectively. In addition, compared with YOLO-VOLO, the results show that YOLO-VOLO-LS has increased 1.451, 3.274, 2.982, and 3.131 in terms of Test-acc, Recall, Precision, and F1-score, respectively. Compared with Slice-VOLO-LS, YOLO-VOLO-LS has increased 5.77, 8.134, 7.69, and 7.915 in terms of Test-acc, Recall, Precision, and F1-score, respectively. After analysis, by combining the advantages of target detection and target classification, not only the background interference is reduced, but also the amount of training target data is increased. Therefore, YOLO-VOLO performed well.

**TABLE 6:** The class activation map (CAM) of VOLO-D1.

Class	V1	V2	V3	V4	V5
SP					
	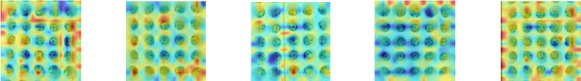				
Day 1					
	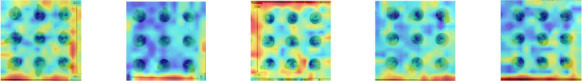				
Day 6					
	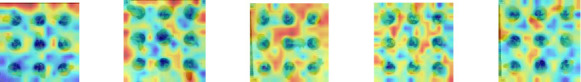				
Day 12					
	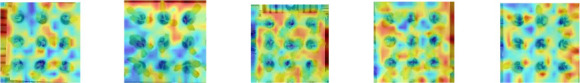				
Day 18					
	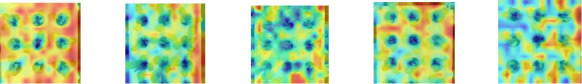				
Day 24					
	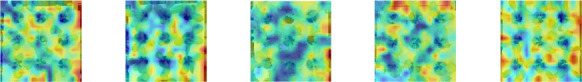				
Day 30					
	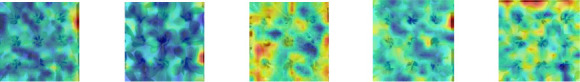				

### Comparison of Modeling Methods for Lettuce Variety Recognition

In order to further prove the performance of our proposed method YOLO-VOLO-LS, we compared the model results with mainstream classification methods such as VGG, ResNet, DenseNet, MobileNet, ShuffleNet, EfficientNet, etc. The specific results are shown in Table 7.

**TABLE 7:** Comparison of modeling methods for lettuce variety recognition.

Class	Image-size	Train-acc	Val-acc	Test-acc	Recall	Precision	F1-score
VGG16	384	99.863	85.861	77.562	86.580	87.685	87.129
ResNet50	384	99.782	73.872	73.846	72.124	74.137	73.117
DenseNet169	384	99.736	81.975	77.254	81.973	85.387	83.645
MobileNet_v2	384	99.936	74.395	70.872	72.108	80.921	76.261
ShuffleNet_v2	384	97.826	72.414	72.441	73.631	78.675	76.069
EfficientNet-B4	384	99.563	73.128	72.340	74.123	85.348	79.341
YOLO-VOLO	384	99.181	**92.542**	**92.001**	**92.785**	**93.032**	**92.908**
YOLO-VOLO-LS	384	99.652	**95.961**	**93.453**	**96.059**	**96.014**	**96.039**

*Bold highlights the results of model comparison.*

**VGGNet** (Simonyan and Zisserman, 2018): VGGNet is a deep CNN proposed in the early stage. Its author is a researcher from Oxford University Computer Vision Group and Google Debug. This method explores the relationship between network depth and model performance by repeatedly stacking 3*3 small convolution kernels and 2*2 maximum pooling layers, and a volume of 16–19 layer CNN is constructed. VGGNet won the runner-up of the ILSVRC 2014 competition and is the champion of the positioning project, with an error rate of 7.5% on the top 5. So far, VGGNet is still used by downstream tasks such as detection and segmentation to extract image features.

**ResNet** (He et al., 2016): The ResNet network is formed by adding residual units through a short-circuit mechanism on the basis of the VGG19 network. Compared with the VGG19 network, the main change of the ResNet network is to directly use the convolution of stride = 2 for downsampling, and use the global average pool layer to replace the fully connected layer. The key design principle of ResNet is that the number of feature maps is doubled when the size of the feature map is reduced by half, which maintains the complexity of the network layer. On the basis of the ResNet 18 network, ResNet 34, ResNet 50, ResNet 101, and ResNet 152 have also been proposed.

**DenseNet** (Huang et al., 2017): DenseNet is a CNN with dense connections between any two layers. The input of any layer of the network is the union of the outputs of all the previous layers. Unlike VGG and Inception, which improve the model in depth and width, respectively, this method starts with features and makes full use of the features of each layer in the network to achieve better model effect and fewer parameters. Therefore, the network not only strengthens the delivery and utilization of features, but also alleviates the influence of gradient disappearance during training process.

**MobileNet** (Howard et al., 2017): MobileNet is a lightweight CNN for embedded intelligent devices. The basic module of the network is the depthwise separable CNN, and then the lightweight network is designed based on the streamlined architecture. Among these, different convolution kernels are used for feature extraction for each input channel through depthwise revolution, and then 1 * 1 convolution check input is used for feature extraction through pointwise revolution, and then the features of the above two steps are fused. In essence, it is similar to the operation process of a standard convolution, but the amount of parameters is greatly reduced. Compared with other popular network models on ImageNet classification, MobileNet shows strong performance.

**ShuffleNet** (Zhang X. et al., 2018): ShuffleNet is a highly efficient CNN architecture specially applied to computer equipment with limited computing power. The architecture uses point-by-point group convolution and channel shuffling operations to use more feature mapping channels within a given computational complexity budget, so as to greatly reduce the amount of calculation while maintaining similar accuracy to the existing advanced models.

**EfficientNet** (Tan and Le, 2019): EfficientNet is a kind of network similar to VGG11-19, ResNet 18–101, wide-resnet 50, 101 networks but different from those proposed by Tan and Le (2019) This network does not arbitrarily scale network dimensions such as depth, width, and resolution like traditional methods, but uses a new model scaling method that uses a series of fixed scale scaling factors to uniformly scale the network dimensions. Through the author’s unremitting efforts and innovation, there are 8 types of networks: EfficentNet-b0, EfficentNet-b1, EfficentNet-b2, EfficentNet-b3, EfficentNet-b4, EfficentNet-b5, EfficentNet-b6, and EfficentNet-b7.

By comparing and analyzing the method proposed by this research with the current mainstream target classification methods, the result shows that the method proposed by this study has significant advantages in Val-acc, Test-acc, Recall, Precision, F1-score, and can effectively solve the problem of classification of lettuce varieties in the SP stage. Based on the similarity between different varieties of lettuce plants in the SP stage, the use of the LS trick also significantly improves the recognition performance of the model.

## Discussion

### Differences in Identification of Lettuce Varieties at Different Growth Stages

In the research process of crop classification, most research mainly focuses on field crop planting scenarios, such as region type, corn, soybeans, barley, spring wheat, dried beans, sugar beets, and alfalfa, mostly supported by remote sensing technology (Zhang H. et al., 2018; Teimouri et al., 2019; Gadiraju et al., 2020; Mazzia et al., 2020; Sun et al., 2020). A few studies have explored crop identification methods from the perspective of leaves or veins, and most of them are supported by visual technology (Grinblat et al., 2016; Bisen, 2021). This research mainly uses the facility greenhouse as the main research scene, combined with deep learning and visual technology to explore the classification methods of small groups of crops near the ground. Therefore, we took 5 kinds of hydroponic lettuce as the research object, the VOLO-D1 method was used to construct a variety classification model for lettuce in different growth stages (see section “Variety Recognition of Lettuce in Multiple Growth Stages Based on VOLO-D1”). The results show that the recognition effect in the SP stage needs to be improved, and the recognition effect in the growth stage after transplanting is very good. In order to further analyze the reasons for this difference, we randomly obtained an image for the lettuce dataset of different growth stages to generate a Class Activation Map for analysis, and the specific results are shown in Table 6.

The result shows that in the SP stage, lettuce plants are small and background interference is large, and most of the attention in the model learning process is background features. After the lettuce is transplanted, during the growth stage, the plants gradually grow, and the individual differences between different varieties are gradually obvious. In addition, as the plant grows, the learning focus of the model gradually shifts from the background to the leaves of the plant, and the interference of the background on the identification of different varieties of lettuce is gradually reduced. The stronger the ability to learn key features, the better the performance of the model phenotype. Facts have proved that the crop recognition classification model with leaves as input is more effective, and this has also been indirectly proved in previous studies (Lee et al., 2017). Therefore, in view of the difficulties in the precise identification of lettuce in the SP stage, we combined the advantages of the target detection mechanism and the target classification mechanism, and we propose a new method of YOLO-VOLO-LS to solve this key problem.

### Selection of Identification Methods for Lettuce Varieties

In the process of constructing the classification model, different methods have different advantages. By comparing the current mainstream target classification methods, the results prove that the method we propose has obvious advantages regardless of the performance of the model itself or the learning focus of the model. In order to further analyze the difference in model performance between different methods, we use the Class Activation Map method to analyze the learning focus of different models, and the specific results are shown in Table 8. The result shows that different methods focus on different points in the model training process. Some methods can only extract part of the image features during the training process. For example, VGG and ResNet mainly extract the edge features of the image. Among them, VGG16 replaces the larger convolution kernel with a continuous 3 * 3 convolution kernel while increasing the network depth (e.g., 11 * 11, 7 * 7, 5 * 5), under the given receptive field conditions, the stacking effect of small convolution kernel is better than that of large convolution kernel (Simonyan and Zisserman, 2018). ResNet adds a direct channel between layers of the network, which effectively avoids the loss of information transmission between layers and reduces the possibility of gradient disappearance or gradient explosion (He et al., 2016). Therefore, ResNet is better than VGG in feature information extraction and retention. Some methods can only extract the central and local features of the image during the training process, such as DenseNet169, MobileNet_v2, ShuffleNet_v2, EfficientNet-B4 mainly focus on the central area of the image, and the edge feature information is lost. Among them, DenseNet169 is different from the previous improvements in network length and width, and in order to make maximum use of the characteristic information between layers, DenseNet169 connects all layers on the premise of ensuring the maximum information transmission between layers, making the network narrower, making fewer parameters and producing a better effect (Huang et al., 2017). ShuffleNet_v2 uses channel splitting to achieve the effect of feature reuse, so as to improve the computational efficiency of the model (Zhang X. et al., 2018). EfficientNet-B4 improves the performance of the model mainly through a synergy coefficient in terms of network depth, width and resolution (. MobileNet_v2 based on the residual block, first uses 1 × 1 lower channel to pass through ReLu, then uses 3 × 3 space convolution to pass through ReLu, and then uses 1 × 1 convolution to recover the channel, which reduces the amount of calculation and improves the performance of the model (Howard et al., 2017). Therefore, MobileNet_v2 is superior to other methods in feature extraction. However, due to the different emphasis of each method, the performance effect of the model may be different. In the actual use process, a method suitable for your own data set is selected through comparative analysis.

**TABLE 8:** The class activation map (CAM) of different methods.

Class	V1	V2	V3	V4	V5
VGG16					
	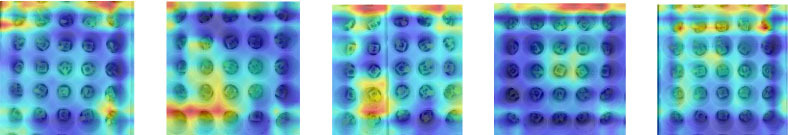				
ResNet50					
	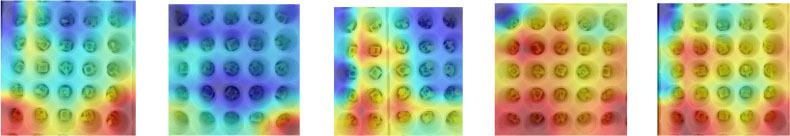				
DenseNet169					
	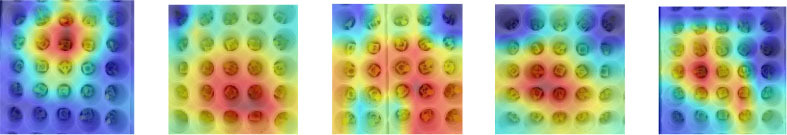				
MobileNet_v2					
	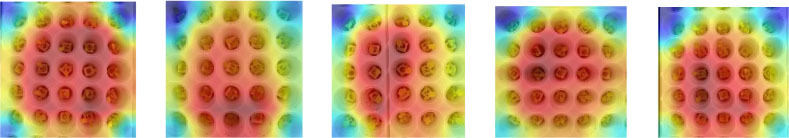				
ShuffleNet_v2					
	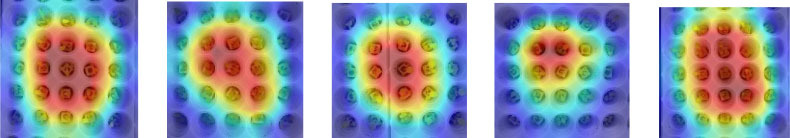				
EfficientNet-B4					
	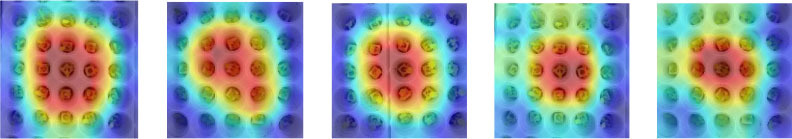				
YOLO-VOLO-LS					
	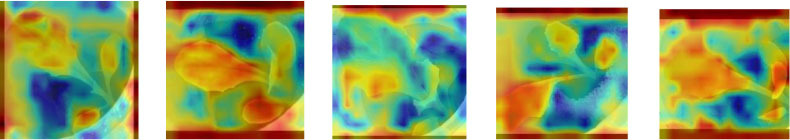				

In this study, a method called YOLO-VOLO-LS is proposed based on analyzing the characteristics of the lettuce dataset in the SP stage, and aimed at solving the problems of small target detection, large background interference, and high individual similarity by combining the advantages of target detection and target classification. Considering the cost of data labeling and the performance of the model, based on the respective advantages of target detection and target classification, we adopted the strategy of first detection and then classification to classify the lettuce in the SP stage. Through this process, we simplified the group target classification problem into an individual target classification problem. While minimizing the influence of the background on the classification of lettuce varieties, the model can learn more leaf details to improve the recognition ability of the model. By observing the Class Activation Maps of YOLO-VOLO-LS and other methods, we can clearly find that our proposed method can almost completely extract the characteristic information of lettuce plants, which is why this method has obvious advantages in accuracy. Similar studies have also proved that crop classification based on the characteristics of leaves or veins has a significant improvement in the model effect (Lee et al., 2017; Tan et al., 2020; Bisen, 2021). This is why our study uses first detection and then classification when classifying lettuce in the SP stage.

In addition, the method we propose plays a role of data enhancement to a certain extent. In order to verify the contribution of increasing the amount of data and reducing the background interference to the SP stage lettuce variety recognition model, we compare the results in section “Variety Recognition of Lettuce During SP Period With a Fusion of YOLO and VOLO.” By slicing the original data set with 5 rows and 5 columns, the data volume can be consistent with the YOLO-VOLO-LS method, and then the VOLO-LS method is used for training verification, and it is found that the improvement of the model performance is very limited. Therefore, we found that removing the background interference to the maximum extent contributes the most to the model, which further verifies the effectiveness of the method proposed in this study. At the same time, during the image slicing process, there is no guarantee that the target in the image is completely segmented, which may cause some plants to be damaged during the slicing process. This may also be the reason for the general performance of the sliced dataset.

Although the method proposed in this study is effective, it still has certain limitations. First, the method proposed in this research is more suitable for the variety identification of small target crops in low-altitude and high-density scenarios such as facility greenhouses. The early variety identification of field crops obtained from high-altitude scenes such as remote sensing has yet to be tried and verified. Second, although the method of first detection and then classification can significantly improve the early variety recognition effect of lettuce seedlings, the specific calculation process may take a long time. Finally, in the follow-up research process, on the one hand, we plan to build a set of software and hardware intelligent detection systems suitable for different growth periods for greenhouse crops based on existing research, and on the other hand, in order to further improve the applicability of this method, we want to apply this method to early crops in field scenarios.

## Conclusion

In this study, a variety identification model was constructed for hydroponic lettuce grown in a greenhouse under the conditions of different growth periods. The results found that the performance of the lettuce variety classification model at the SP stage before the lettuce transplantation still needs to be improved. By combining the respective advantages of the target detection mechanism and the target classification mechanism, we innovatively propose a classification method for lettuce varieties at the SP stage, called YOLO-VOLO-LS. This method has achieved excellent results of 95,961, 93,452, 96,059, 96,014, 96,039 in Val-acc, Test-acc, Recall, Precision, and F1-score, respectively. In addition, we have achieved nearly 100% of the lettuce classification effect in the growth stages of Days 1, 6, 12, 18, 24, and 30 by adopting the VOLO-D1 method. In view of the characteristics of lettuce seedlings in the SP stage, we simplified the group classification problem to an individual classification problem by adopting the strategy of first detection and then classification, which significantly improved the performance of the model. Of course, this method may be more suitable for research on the variety identification of high-density small target crops in a low-altitude environment. The small target image of the group can be cropped through the detection method, which not only increases the amount of data, but also reduces the background interference. Therefore, through the combination of detection and classification methods, on the one hand, the problems of small target crop similarity and background interference can be overcome, and on the other hand, the problem of small samples can be solved to a certain extent, which has a certain contribution in data preprocessing.

## Data Availability Statement

The raw data supporting the conclusions of this article will be made available by the authors, without undue reservation.

## Author Contributions

PZ collected literature and training models together and completed the manuscript writing. DL modified the manuscript. Both authors read and approved the final manuscript.

## Conflict of Interest

The authors declare that the research was conducted in the absence of any commercial or financial relationships that could be construed as a potential conflict of interest.

## Publisher’s Note

All claims expressed in this article are solely those of the authors and do not necessarily represent those of their affiliated organizations, or those of the publisher, the editors and the reviewers. Any product that may be evaluated in this article, or claim that may be made by its manufacturer, is not guaranteed or endorsed by the publisher.
