# Supplementary material for: YOLO-VOLO-LS: A Novel Method for Variety Identification of Early Lettuce Seedlings
Source: Front Plant Sci. 2022 Feb 24;13:806878. doi: 10.3389/fpls.2022.806878 (PMC8909383; doi:10.3389/fpls.2022.806878)
Supplement: Supplementary file 1 [file Table_1.docx]

Supplementary Material

First, after we separated the 5 varieties of lettuce from the seeding cotton, we collected data every other day for the next 12 days. We collected 6 times in total, and 160 images of each lettuce are collected each time. Second, after the 5 varieties of lettuce seedlings were transplanted to the stereo cultivation rack, we carried out image data acquisition every 5 days. Each data collection mainly acquires 50 lettuce images of different varieties and different nitrogen nutrient gradient treatments, each of which has 6 nitrogen treatments, for a total of 300 images.

**TABLE 1 | Time arrangement of image data acquisition**

| Class | SP (12 days) | Day 1 | Day 6 | Day 12 | Day 18 | Day 24 | Day 30 |
| --- | --- | --- | --- | --- | --- | --- | --- |
| V1 | 960 | 300 | 300 | 300 | 300 | 300 | 300 |
| V2 | 960 | 300 | 300 | 300 | 300 | 300 | 300 |
| V3 | 960 | 300 | 300 | 300 | 300 | 300 | 300 |
| V4 | 960 | 300 | 300 | 300 | 300 | 300 | 300 |
| V5 | 960 | 300 | 300 | 300 | 300 | 300 | 300 |
